# Faster and more accurate patient positioning with surface guided radiotherapy for ultra-hypofractionated prostate cancer patients

**DOI:** 10.1016/j.tipsro.2021.07.001

**Published:** 2021-09-04

**Authors:** Annika Mannerberg, Malin Kügele, Sandra Hamid, Anneli Edvardsson, Kristoffer Petersson, Adalsteinn Gunnlaugsson, Sven Å.J. Bäck, Silke Engelholm, Sofie Ceberg

**Affiliations:** a Department of Medical Radiation Physics, Lund University, Lund, Sweden; b Department of Hematology, Oncology and Radiation Physics, Skåne University Hospital, Lund, Sweden; c Department of Oncology, Oxford Institute for Radiation Oncology, University of Oxford, Oxford, United Kingdom

**Keywords:** Surface guided radiotherapy, Patient setup time, Patient positioning, Radiotherapy workflow

## Abstract

**Introduction:**

The aim of this study was to evaluate if surface guided radiotherapy (SGRT) can decrease patient positioning time for localized prostate cancer patients compared to the conventional 3-point localization setup method. The patient setup accuracy was also compared between the two setup methods.

**Materials and methods:**

A total of 40 localized prostate cancer patients were enrolled in this study, where 20 patients were positioned with surface imaging (SI) and 20 patients were positioned with 3-point localization. The setup time was obtained from the system log files of the linear accelerator and compared between the two methods. The patient setup was verified with daily orthogonal kV images which were matched based on the implanted gold fiducial markers. Resulting setup deviations between planned and online positions were compared between SI and 3-point localization.

**Results:**

Median setup time was 2:50 min and 3:28 min for SI and 3-point localization, respectively (*p* < 0.001). The median vector offset was 4.7 mm (range: 0–10.4 mm) for SI and 5.2 mm for 3-point localization (range: 0.41–17.3 mm) (*p* = 0.01). Median setup deviation in the individual translations for SI and 3-point localization respectively was: 1.1 mm and 1.9 mm in lateral direction (*p* = 0.02), 1.8 and 1.6 mm in the longitudinal direction (*p* = 0.41) and 2.2 mm and 2.6 mm in the vertical direction (*p* = 0.04).

**Conclusions:**

Using SGRT for positioning of prostate cancer patients provided a faster and more accurate patient positioning compared to the conventional 3-point localization setup.

## Highlights

• 1 min time reduction for prostate patient setup using SGRT.
• Accurate initial positioning for deep-seated target with surface imaging.
• Improved workflow using intuitive color map for setup guidance.

## Introduction

Image guided radiotherapy (IGRT) is used in modern radiotherapy to minimize setup errors due to both inter- and intrafractional patient and tumour motion [1], [2]. Recently, a surface imaging (SI) modality has been adopted into the IGRT toolbox with the potential to further decrease the effect of inter- and intrafractional motion during patient positioning and treatment delivery [3], [4]. Surface guided radiotherapy (SGRT) generates real-time three-dimensional (3D) surface images of the scanned patient, which is compared to a reference surface for positioning purposes. Unlike the simple conventional 3-point localization setup method, SGRT provides additional information of the patient topography, highlighting patient posture errors and anatomical deformations (such as swelling or weight loss) [5]. As SI does not contribute to any radiation exposure, it can be used for patient monitoring during treatment delivery. Also, SGRT has the capability of automatic beam-hold if the patient motion exceeds a pre-set threshold [4]. Previous studies have shown that SGRT can provide accurate positioning for various treatment sites and treatment techniques [3], [6], [7], [8], [9], [10]. Most widespread, SI has been clinically implemented for positioning of breast cancer patients since the target position is well represented by the surface [8], [11], [12]. The improved setup has the potential to decrease the amount of verification images, which could reduce both setup time and absorbed dose to the patients [4], [8], [13]. For internal treatment sites, the target position is not always well represented by a surface image, resulting in reduced positioning accuracy [3], [6], [14]. For targets in abdomen and pelvis, SGRT achieves the similar accuracy as 3-point localization and is often considered to be used as a complement to verification images [3], [6], [14].

For prostate cancer radiotherapy, the treatment time should preferably be kept as short as possible due to the increased risk of prostate motion over time [15], [16], [17]. Having an accurate and fast patient positioning is therefore of particular importance. In recent years, ultra-hypofractionation with a high fractional dose delivered with flattening filter free (FFF) volumetric modulated arc therapy (VMAT) in few fractions has been proposed and implemented in our clinic [18]. With fewer fractions, it is less likely for the total delivered dose distribution to be evenly blurred around the target due to setup deviations. Hence, accurate patient positioning is even more crucial for ultra-hypofractionated treatment. To minimize potential setup errors, the initial patient setup method should provide a reliable correlation to the treatment position. Further, only small shifts after verification imaging are preferable to minimize patient displacements caused by large couch shifts. Two early publications have shown SGRT to be a reproducible and non-invasive method for positioning of prostate cancer patients treated with 3D-conformal radiotherapy [19], [20]. Only a few studies have examined the time efficiency of using surface imaging during patient positioning instead of 3-point localization setup [9], [10]. These studies showed that the total treatment time can be reduced while improving or maintaining patient position accuracy, however, both studies were carried out on a TomoTherapy treatment system using the time consuming megavoltage computed tomography (MVCT) for image guidance.

The aim of this study was to investigate if SGRT could improve the setup workflow by reducing the setup time while maintaining the positioning accuracy for prostate patients receiving ultra-hypofractionation FFF-VMAT treatment. To our knowledge this is the first study to examine if SGRT can reduce the patient setup time for prostate cancer patients.

## Method

### Ethics

The Regional Ethical Review Board in Lund has approved retrospective research of radiotherapy data (No. 2013/42).

### Patients

A total of 40 localized prostate cancer patients were included in this study. Each patient received 7 fractions of a 6 MV FFF ultra-hypofractionated VMAT treatment plan with a total absorbed dose of 42.1 Gy, delivered with a TrueBeam linear accelerator (ver 2.5, Varian Medical Systems, Palo Alto, CA). All patients had a CTV to PTV margin of 7 mm, in line with the HYPO-RC-PC trial [18]. The CTV included the prostate only and all patients had three gold fiducial markers implanted at least two weeks prior to start of radiotherapy treatment.

### Positioning

Twenty patients were positioned using the conventional 3-point localization setup method, where skin tattoos were aligned with in-room lasers. The remaining 20 patients were positioned with SI setup where a single camera Catalyst™ (C-Rad Positioning AB, Uppsala, Sweden) system was used for positioning. In this study, patient positioning refers to the initial setup carried out prior to acquisition of verification images.

The beginning of positioning was the same for all patients. All patients were positioned in a Combifix™ (Civco Radiotherapy, IA, USA) for fixation of the knees and legs and were holding a small ring in their hands, placed on their chest. During the first fraction the in-room lasers were aligned with the patient’s tattoos. In order to move the patient from the reference position to the isocenter position, a manual couch shift was performed by the radiation therapists (RTTs). When in isocenter position, marks aligning with the lasers was drawn on the patient’s skin.

For all patients, the position was always verified using orthogonal kilovoltage (kV) images, which were considered to be the gold standard. The fiducial markers in the kV images were matched to the position in the digital reconstructed radiograph (DRR). The deviations obtained in lateral (lat), longitudinal (lng) and vertical (vrt) directions from the image matching were compared between SI and 3-point localization setup. The total vector offset, *v*, was calculated using Eq. (1).

(1) $$v=\sqrt{\mathrm{la}t^{2}+lng^{2}+vrt^{2}}$$

In total, 280 paired images were analysed regarding patient positioning accuracy.

For the patients positioned with 3-point localization the in-room positioning was considered to be complete when the lasers were aligned with the patient’s skin marks. The RTTs then left the treatment room and proceeded with the acquisition of the orthogonal kV images to verify the position of the gold fiducial markers.

#### Surface imaging setup

The optical surface scanning system Catalyst™ creates 3D surface images for patient positioning. The hardware and functionality of the Catalyst™ system has been described elsewhere [6], [8].

Skin marks were drawn during the first fraction for patients positioned with SI as well. This was done to help the RTTs in the transition to the new positioning method and these markers were often used the following fractions as a quick initial check for rotations. For SI setup, the reference surface used was the body structure of the planning CT data set, imported from the treatment planning system. Before treatment start, genitalia and the most cranial part of the stomach was cropped from the reference surface.

**Fig. 1:**
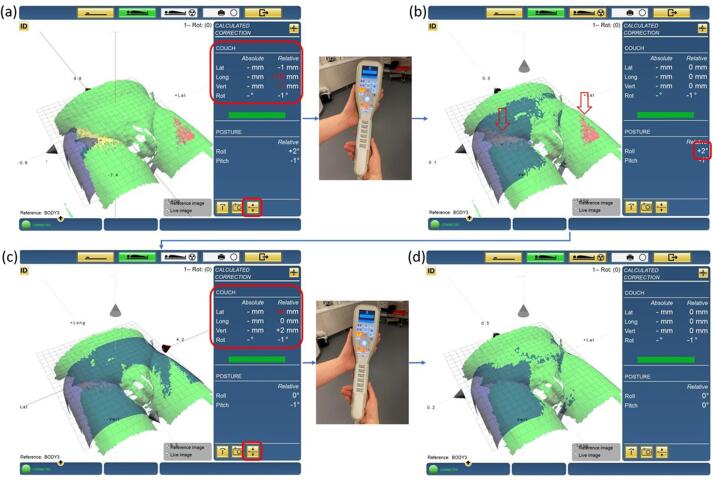
A surface imaging example. The blue and green surfaces are the reference and live surfaces, respectively. The couch was initially shifted to isocenter position using saved couch parameters. The shift indicated by Catalyst™ (Lat −1 mm, Lng + 10 mm, Vrt −7 mm) (a) was then applied using the Auto-GoTo function. The color map and the positioning result indicated a roll (b), which was corrected for by asking the patient to adjust himself. Once the roll was corrected for, residual translations (Lat + 4 mm, Lng 0 mm, Vrt + 2 mm) (c) were applied using Auto-GoTo into the correct treatment position (d). (For interpretation of the references to color in this figure legend, the reader is referred to the web version of this article.)

To obtain the best possible live surface with good surface coverage of the patient, the settings of the Catalyst™ were optimized during the first fraction. Thereafter, the RTTs corrected for any rotations by comparing the live and reference surfaces and using a color map projected on the patient. This color map is a live feedback of the patient position and shows if the live position of the patient differs from the reference one in red and green color. A tolerance level for deviations between live and reference surface can be set, and only deviations larger than this threshold will activate the color map projection [[8]]. In this study, the color map threshold was set to 5 mm. Lastly, the couch was shifted with Auto-GoTo to the correct isocenter position calculated with the non-rigid algorithm of the Catalyst™ system. When pressing the Auto-GoTo button, the couch coordinates for positioning the patient in isocenter are sent from the Catalyst™ system to ARIA (Varian Medical Systems, Palo Alto, USA). After this, a button on the treatment couch pendant can be pressed and the couch is automatically moved in lat, lng and vrt according to the calculations done by the Catalyst™ (Fig. 1).

During the following fractions, the patient was positioned on the couch and the RTTs quickly checked the lasers and skin marks. The couch was initially shifted to the isocenter position using the Auto-GoTo function (Fig. 1) using the saved couch parameters. Thereafter, also using the Auto-GoTo function, the couch was shifted to the correct isocenter position according to the Catalyst™. The live and reference surfaces were compared, and any rotations indicated by the Catalyst™ were corrected for. In presence of pelvis rotation, the color map was projected onto the patient’s skin for setup correction guidance for the RTTs. The RTTs asked the patient to lift his hips and turn in the direction instructed by the red and yellow colors. After such correction, the Auto-GoTo was used to correct for any residual translations (Fig. 1).

### Setup time

The setup times were retrieved from system log files in ARIA. The start of positioning was defined as when the RTTs turned the in-room lasers on or when they first started moving the couch after the patient had been opened in ARIA for treatment, whichever occurred first. The end of positioning was when image acquisition started.

In our clinic there is no separate initial setup session prior to treatment start and instead all initial setup is carried out during the first treatment fraction. During the first fraction of SI setup, the RTTs went through all the steps that also were carried out during the first fraction of 3-point localization setup. This entailed correcting for rotations using the 3-point skin tattoos, manually shifting the couch from reference position to isocenter position and drawing skin marks in the isocenter position, before using SI for positioning. Thus, the time spent on 3-point localization and SI could not be resolved. The positioning time of the first fraction for SI setup was therefore not considered representative and was excluded. To obtain a fair positioning time comparison between the two different setup methods, the setup time of the first fraction for 3-point localization was also excluded. The setup time was investigated for 240 fractions.

### Statistics

The setup time and setup deviation distributions were tested for normality using the Shapiro Wilks test. Setup times were not normally distributed. Consequently, the Mann-Whitney *U* test was used for comparing the setup times for the two methods. Positioning deviations in the lng and vrt direction for SI were normally distributed, however, positioning deviations for 3-point localization were not normally distributed. To test the hypothesis that the two setup methods result in equal setup accuracy, a Mann-Whitney *U* test was carried out. A significance level of α = 0.05 was used for all tests.

## Results

### Setup time

**Fig. 2:**
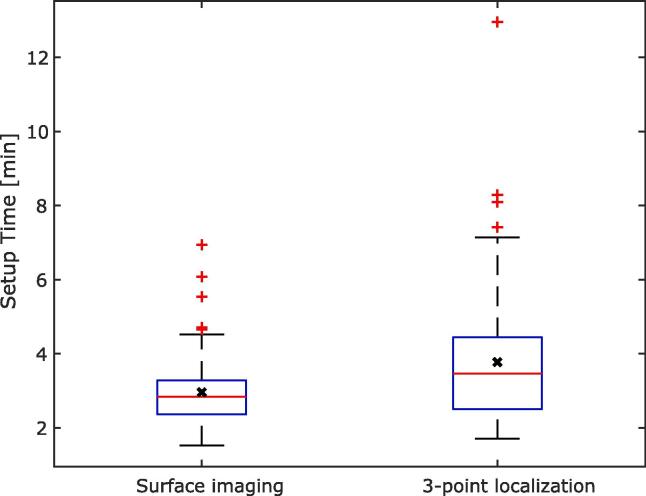
Comparison of patient setup time for surface imaging and 3-point localization setup. The lower quartile is the 25th percentile and the upper quartile is the 75th percentile. The red horizontal lines respresent the median setup time, the black crosses show the mean setup time. The whiskers shows the nonoutlier minimum and maximum value. Outliers are values larger than 1.5 times the interquartile range and are displayed as red plus signs. (For interpretation of the references to color in this figure legend, the reader is referred to the web version of this article.)

The median setup time was 2:50 min (min) (range: 1:32–6:56 min) for SI, and 3:28 min (range: 1:42–12:57 min) for 3-point localization (*p* < 0.001) (Fig. 2). On average the setup time decreased with 49 s for each fraction, using SI (Fig. 2).

### Positioning

**Fig. 3:**
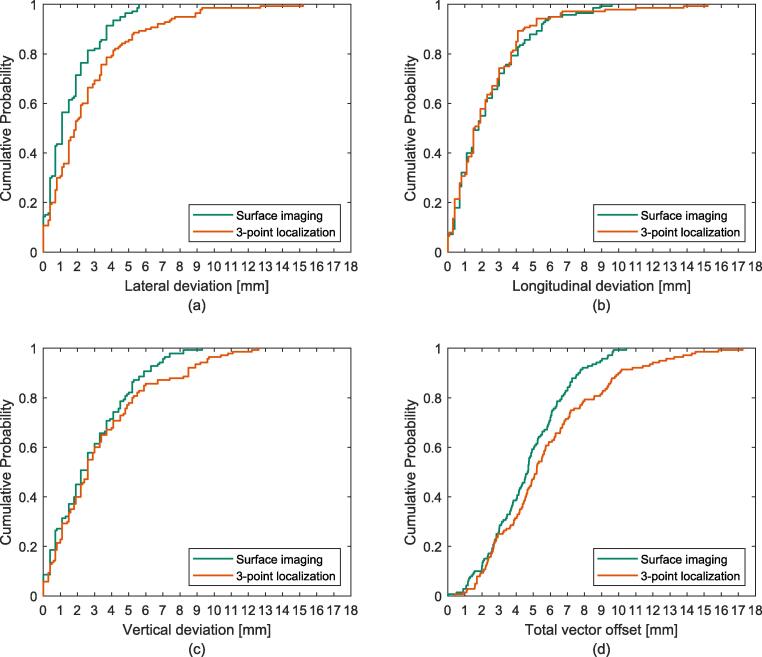
The cumulative probability for setup deviation comparing surface imaging and 3-point localization setup in the lateral (a), longitudinal (b) and vertical (c) direction as well as the total vector offset (d), verified with orthogonal kV images.

The median setup deviation in the lat translation was 1.1 mm (range: 0–5.6 mm) for SI and 1.9 mm (range: 0–15.2 mm) for 3-point localization (*p* = 0.02) (Fig. 3a). For lng setup deviations the median was 1.8 mm (range: 0–9.6 mm) for SI and 1.6 mm (range: 0–15.2 mm) (*p* = 0.41) (Fig. 3b). For vrt the median setup deviation was 2.2 mm (range: 0–9.3 mm) for SI and 2.6 mm (range: 0–12.6 mm) for 3-point localization (*p* = 0.04) (Fig. 3c).

The median vector offset was 4.7 mm (range: 0–10.4 mm) for SI and 5.2 mm (range: 0.41–17.3 mm) for 3-point localization (*p* = 0.01). The probability of positioning a patient within a total vector offset of 7 mm was 84% for SI and 71% for 3-point localization (Fig. 3d). For 90% of the fractions the total vector offset was within 7.6 mm for SI and 10.1 mm for 3-point localization. For SI, only one fraction had a total vector offset larger than 10 mm, which implies that such large setup deviations occurs less than every 100th fraction. However, for 3-point localization a setup deviation larger than 10 mm occurred approximately every 10th fraction.

## Discussion

In this study, the potential of SGRT for ultra-hypofractionated prostate cancer radiotherapy treatment, in terms of patient setup efficiency and accuracy, was investigated. Large setup deviations were reduced, while patient setup time was improved with 20% using SI. These findings are to our knowledge the first to show improved setup efficiency while maintaining the standard daily IGRT prostate protocol. It is of great importance to reduce the total treatment time for prostate cancer patients since prostate motion increases over time [15], [16], [17]. Langen et al. [15] showed that the prostate can drift 5 mm from isocenter after only 4 min and Ballhausen et al. [16] showed that the variance in prostate position increase over time for all prostate cancer patients. Even small total treatment time reductions in the order of minutes can therefore lead to a reduced effect of intrafractional motion, which is especially important for patients treated with a high fraction dose. Further, a shorter treatment time could also result in higher patient comfort and the possibility to treat more patients. Previously, FFF beams have been implemented which has halved the beam on time [21] for prostate cancer radiotherapy treatments.

This study was designed to isolate the initial setup time between SI and 3-point localization. It is therefore certain that time reductions shown in this study can be traced to the use of SGRT only. The results are independent of for instance the IGRT method used or different treatment techniques. Hence, other clinics who choose to start positioning patients with SI can expect around 1 min in time reduction per treatment fraction.

The patient setup time was found to be significantly reduced using SGRT for positioning of prostate cancer patients, which shows that SGRT is a potent tool to further reduce the treatment fraction time, by reducing the patient setup time. The reduced setup time could be because of a standardized workflow for surface imaging. Thus, the steps in the software are rigid and leaves no room for manual couch adjustments. The information from the color map mitigates the RTTs’ subjectivity on how well the patient needs to be aligned since the color map and rotations must be fulfilled, hence the SI system works as an operator-independent check for the patient setup.

When this study was conducted, the use of SI for prostate cancer patients had just been started. The RTTs was therefore at the beginning of their learning curve on how to use the Catalyst™ system for these patients. The Auto-GoTo function was introduced in connection with this project and was an additional step in the SI workflow to learn. Further time reductions might be achievable due to increased experience of using the SGRT system. As a further consequence of this study, the RTTs have omitted drawing skin marks onto the patients, which also contributes to reducing the setup time.

Previous studies [3], [6], [14] have not shown any improvement using SGRT for pelvis positioning. This could be due to the fact that in those studies, they grouped different target sites in the most caudal part of the body into pelvis/lower extremities. To our knowledge this study is the first to investigate positioning accuracy for prostate patients receiving ultra-hypofractionation FFF-VMAT treatments. In the study by Stanley et al. [3], the average vector offset for treatment sites in the pelvis/lower extremities was 6 mm for setup using surface scanning. This agrees well with the median vector offset obtained in our study of 4.7 mm for SI. However, Stanley et al. found that the average vector offset for setup with lasers and skin marks for pelvis/lower extremities was 9 mm. This is slightly higher than the results in this study where the median vector offset was 5.2 mm for 3-point localization.

An early study by Bartoncini et al. [20] showed improved patient setup in lat and vrt directions using a different SI system and bony image registration for prostate patients. Bartoncini et al. evaluated if SI correlated to verification image registration and did not compare SI to the conventional 3-point localization setup. Hence, the present study is the first to show an improvement in the setup workflow and patient positioning. Positioning results in this study, showed that SI provides a significantly improved surrogate for the target in lat and vrt directions compared to 3-point localization. The improvement in the lat direction could be explained by the fact that a single central tattoo is used for 3-point localization, whereas the full topography of the patient is used for the SI setup. Moreover, while the non-normal distribution of setup deviations for 3-point localization implies a subjective setup method, the normally distributed setup deviations for SI are another indication of a more operator-independent method. Further, a single camera Catalyst™ system was used for setup in this study. However, with a 3-camera Catalyst™ system the patient setup accuracy could potentially be further improved. Additionally, based on these results, patient positioning using SI could potentially be applied for other deeply seated targets.

We have found SI to be useful in combination with kV imaging to prevent patient setup deviations prior to verification imaging. SGRT can be considered as an additional safety component in case imaging is left out or for target sites where daily images are not acquired.

Although outside the scope of this study, another important finding was the improved physical work environment reported by the RTTs when using SI for prostate cancer patients. When SI was used there was a lot less hands-on work for the RTTs and there was not as much need for lifting and pushing the patient into the correct position. Since the start of this project the RTTs have therefore reportedly experienced a reduction in the amount of back and shoulder pain. These results are however not part of this study and should be further investigated.

## Conclusion

Surface guided radiotherapy with the Catalyst™ system reduced the patient setup time by approximately 1 min per treatment fraction. Additionally, the initial patient setup accuracy was significantly improved using the surface imaging setup method prior to IGRT.

## Declaration of Competing Interest

The authors declare the following financial interests/personal relationships which may be considered as potential competing interests: [Our research is partly financially supported by C-Rad AB, Uppsala, Sweden, however the authors alone are responsible for the content, analyses and writing.].
